# Comparative Evaluation of Data Dependent and Data Independent Acquisition Workflows Implemented on an Orbitrap Fusion for Untargeted Metabolomics

**DOI:** 10.3390/metabo10040158

**Published:** 2020-04-18

**Authors:** Pierre Barbier Saint Hilaire, Kathleen Rousseau, Alexandre Seyer, Sylvain Dechaumet, Annelaure Damont, Christophe Junot, François Fenaille

**Affiliations:** 1 Département Médicaments et Technologies pour la Santé (DMTS), CEA, INRAE, Université Paris-Saclay, MetaboHUB, F-91191 Gif sur Yvette, France; pierre.barbiersainthilaire@gmail.com (P.B.S.H.); kathleen.rousseau@cea.fr (K.R.); annelaure.damont@cea.fr (A.D.); christophe.junot@cea.fr (C.J.); 2 MedDay Pharmaceuticals SA, 24 Rue de la Pépinière, F-75008 Paris, France; alexandre.seyer@medday-pharma.com (A.S.); sylvain.dechaumet@medday-pharma.com (S.D.)

**Keywords:** metabolomics, high resolution mass spectrometry, Orbitrap Fusion, DDA, DIA

## Abstract

Constant improvements to the Orbitrap mass analyzer, such as acquisition speed, resolution, dynamic range and sensitivity have strengthened its value for the large-scale identification and quantification of metabolites in complex biological matrices. Here, we report the development and optimization of Data Dependent Acquisition (DDA) and Sequential Window Acquisition of all THeoretical fragment ions (SWATH-type) Data Independent Acquisition (DIA) workflows on a high-field Orbitrap Fusion^TM^ Tribrid^TM^ instrument for the robust identification and quantification of metabolites in human plasma. By using a set of 47 exogenous and 72 endogenous molecules, we compared the efficiency and complementarity of both approaches. We exploited the versatility of this mass spectrometer to collect meaningful MS/MS spectra at both high- and low-mass resolution and various low-energy collision-induced dissociation conditions under optimized DDA conditions. We also observed that complex and composite DIA-MS/MS spectra can be efficiently exploited to identify metabolites in plasma thanks to a reference tandem spectral library made from authentic standards while also providing a valuable data resource for further identification of unknown metabolites. Finally, we found that adding multi-event MS/MS acquisition did not degrade the ability to use survey MS scans from DDA and DIA workflows for the reliable absolute quantification of metabolites down to 0.05 ng/mL in human plasma.

## 1. Introduction

Metabolomics aims at studying the whole set of metabolites found in a given biological system. Its objectives are therefore to identify and quantify all the small molecules present in complex matrices as comprehensively as possible. Nowadays, liquid chromatography coupled to high-resolution mass spectrometry (LC/HRMS) represents the most widely used approach for untargeted metabolomics [1]. LC/HRMS-based metabolomics workflows usually start with the acquisition of high-resolution full scan accurate mass spectra in order to obtain comprehensive metabolic profiles thanks to the detection of a few thousand signals in a semi-quantitative fashion [2]. The recorded signals then need to be annotated and corresponding metabolites identified to translate raw analytical data into meaningful biological information. However, a large portion of the detected signals remain structurally uncharacterized and thus, metabolite annotation and identification still represent major bottlenecks in untargeted metabolomics [3,4].

The successful identification of metabolites requires a combination of information lines exploited in conjunction, including accurate LC retention time, high mass measurement accuracy (<1 ppm), isotope pattern and isotope fine structure measurement as well as high-quality fragmentation spectra [5,6]. High mass resolution (>100,000) allows for the measurement of isotope pattern and isotope fine structure (through the distinction of isobaric isotopes), which is particularly useful for confident compound annotation [6]. One of the most valuable elements to confirm metabolite annotation or reduce the list of possible annotations is the acquisition of fragmentation spectra and their comparison to reference MS/MS spectra included in spectral libraries [7]. 

Experimental approaches to acquire MS/MS data are generally referred to as Data Dependent or Data Independent Acquisition (DDA and DIA, respectively). In the most widely used DDA acquisition workflow, MS/MS spectra are automatically collected for precursor ions whose MS intensity exceeds a pre-defined threshold. In this mode, precursor ions are selected using a small isolation window (typically ≤ 1 Da wide), which leads to high-quality and higher purity MS/MS spectra. Nevertheless, the selection of precursor ions is a (semi)stochastic event suffering from low analytical reproducibility and favoring selection of the most abundant but sometimes biologically irrelevant ions. As a consequence, some biologically relevant precursor ions may not be selected for fragmentation if they do not match the defined selection criteria or if they are coeluted with more intense species. These issues can be partially overcome by the use and careful optimization of additional MS-to-MS/MS triggering criteria (e.g., isotope pattern or mass defect) [8,9,10], by optimizing the use of exclusion or inclusion lists prior to further MS/MS acquisitions [11,12,13], or by implementing efficient software tools for assessing precursor ion purity of acquired data [14]. 

Until very recently, the implementation of acquisition protocols combining MS and MS/MS acquisitions in one single run, especially those related to DIA, were rather limited to hybrid quadrupole-time-of-flight (Q-TOF) instruments due to their enhanced duty cycles [15,16]. Because of their lower acquisition speed, simultaneous MS and MS/MS data collection can rarely be performed at the highest mass resolution achievable on Orbitrap-based instruments, in order to keep enough data points per chromatographic peak, which is mandatory for further reliable peak integration and corresponding metabolite quantification from MS data [17]. It is therefore a rather common procedure in metabolomics, when using an Orbitrap mass analyzer, to acquire MS/MS data by DDA or targeted-DDA on pooled quality control (QC) samples at the beginning or at the end of an analytical batch [17,18]. Collected data can then be used to support metabolite annotation in biological samples, assuming that the ions fragmented in the pooled QC samples are identical to those present in each of the biological samples analyzed. While separating metabolite quantification from identity confirmation, this approach also requires additional acquisition time and sample volumes.

A DIA acquisition method would represent a viable alternative or complement to DDA to obtain valuable MS/MS data on all ions from all samples for further metabolite structure confirmation and quantification [19]. SWATH (accounting for Sequential Window Acquisition of all THeoretical fragment-ion spectra) [20], which is the most used DIA acquisition workflow, involves the selection of wide and consecutive *m/z* isolation windows (typically between 10 and 50 Da) to cover the whole mass range. All ions within these windows are then fragmented simultaneously. Due to the width of isolation windows, the direct link between a specific precursor ion and its corresponding fragments is often lost, which renders MS/MS data interpretation of unknown metabolites much more difficult than that of MS/MS data resulting from DDA protocols. All-Ion Fragmentation (AIF) is another form of DIA acquisition that has been successfully applied to metabolomics or lipidomic approaches [21,22,23]. The present paper dealing only with SWATH-like DIA as applied to low-mass metabolites (essentially below 700 Da), DIA accounts hereafter for SWATH-like approaches. Although some software solutions for handling MS/MS data obtained from DIA as well as DDA workflows have been published for metabolomics such as MS-DIAL [24] or MetaboDIA [25], no comparative study has been carried out to objectively assess the advantages and limitations of these different tools.

While DIA strategies such as SWATH are often used in proteomics, very limited work has been reported on small molecules [26]. This particularly holds true when considering the use of the Orbitrap mass analyser, with very sporadic examples published [27,28]. In this context, the aim of this study was to evaluate the performance of DDA and SWATH-type DIA acquisition workflows when implemented on an Orbitrap Fusion^TM^ Tribrid^TM^ mass spectrometer. We especially exploited the versatility of this mass spectrometer, comprising of quadrupole, linear ion trap and Orbitrap mass analyzers, in order to conciliate multiple modes of analysis and experimental flexibility. A set of 47 well defined standard compounds was first used for the development and preliminary evaluation of DDA and DIA strategies. Optimized methods were then used to study plasma metabolome using another set of 72 endogenous metabolites. Although reversed-phase liquid chromatography (RPLC) and hydrophilic interaction liquid chromatography (HILIC) provide complementary information and thus represent a relevant combination for expanding metabolome coverage [29,30], we decided to focus this study on RPLC, which is the most demanding in terms of MS-MS/MS acquisition speed as a result of the narrower chromatographic peaks, using routine workflows as used in our laboratory [29,31]. This report demonstrates the value and complementarity of DDA and SWATH-type DIA approaches for metabolomics, as implemented on an Orbitrap Fusion^TM^.

## 2. Materials and Methods

### 2.1. Chemicals and Reagents

Chemical standards were from Sigma-Aldrich (Saint Quentin Fallavier, France). Liquid chromatography solvents, i.e., acetonitrile (ACN) and water, and extraction solvent (i.e., methanol) were LC-MS grade solvents (LC-MS Chromasolv, Sigma-Aldrich, Saint Quentin Fallavier, France). Mobile phase additive (i.e., formic acid) was from Sigma Aldrich. NIST SRM-1950 reference plasma was ordered from the National Institute of Standards and Technology (NIST, Gaithersburg, MD, USA).

### 2.2. Preparation of Standard Samples and Plasma Extracts

A pool of 47 chemical compounds (20 µg/mL for each compound) was prepared from solutions at 1 mg/mL (Supplementary Material, Table S1). The pool was then diluted in water:ACN (95:5) with 0.1% formic acid to reach 100, 10, 3, 1, 0.5, 0.25, 0.1, and 0.05 ng/mL. 

NIST SRM-1950 reference human plasma was processed using a metabolomics-designed extraction adapted from a previously published one [29]. Briefly, 100 µL of plasma were diluted with 400 µL of methanol, sonicated for 15 min and centrifuged at 15,000× *g* for 15 min. After a 1h30 slow precipitation on ice, samples were centrifuged at 20,000× *g* for 15 min and supernatant was withdrawn and dried under nitrogen. Plasma metabolites were resuspended in water/ACN (95:5) with 0.1% formic acid. Some plasma samples were spiked post-extraction with the pool of 47 compounds at a final concentration ranging from 0.05 to 100 ng/mL for each metabolite. 

### 2.3. LC/HRMS Analysis 

Chromatographic separation was performed on a Dionex Ultimate 3000 (ThermoFisher Scientific, Courtaboeuf, France) with a Hypersil Gold C_18_ column (150 × 2.1 mm, 1.9 µm; ThermoFisher Scientific) under gradient conditions, using mobile phases A (0.1% formic acid in water, *v*/*v*) and B (0.1% formic acid in ACN, *v*/*v*). Flow rate was set at 500 µL/min. Mobile phase A was held at 95% for 2 min followed by an increase to 100% of mobile phase B over 11 min. These proportions were kept constant for 12.5 min before returning to 5% B for 4.5 min. Injection volume was set at 5 µL.

Mass spectrometry analyses were performed on an Orbitrap Fusion^TM^ instrument (ThermoFisher Scientific, Courtaboeuf, France) fitted with a HESI source. Source parameters were set as follows: spray voltage at + 3500 V, sheath gas at 50 (arbitrary units), auxiliary gas at 12 (arbitrary units), sweep gas at 3 (arbitrary units), ion transfer tube temperature at 325 °C, and vaporizer temperature at 350 °C. Ion transfer parameters were set as follows: mass range at “normal”, S lens RF level at 60%. Scan parameters applied were as follows: acquisition time from 0 to 20 min, positive ionization mode, micro scan at “1”, data type at “profile”, AGC target at 5 × 10^4^. For acquisition in MS mode only, the data were acquired using the Orbitrap analyzer, at a resolution of 240,000 at *m/z* 200 (FWHM, a spectrum collected at this resolution requires ~0.5 s); maximum injection time was set at 400 ms, and the scan range from *m/z* 85 to *m/z* 1000. More detailed information regarding experimental conditions is given in the Supporting Information (Tables S2–S4).

### 2.4. Data Processing and Evaluation of the Quality of MS/MS Spectra

Raw instrument data (.RAW) were processed using Xcalibur v.2.2.44 (ThermoFisher Scientific) processing methods. The genesis algorithm was used for detection (nearest RT, minimum peak height (S/N) = 3) and integration (smoothing point = 1, S/N threshold = 0.5, enable valley detection = no, constrain peak width = no). Accurate [M+H]^+^ *m*/*z*, fragment ion *m/z* and expected retention time (min) are shown in supplementary data (Supplementary Material, Table S1).

MS-DIAL software [24] (version 4.12) which is freely available (http://prime.psc.riken.jp/compms/msdial/main.html), was used to calculate MS/MS similarity between the evaluated and reference spectra. We merged all the reference MS/MS spectra from our in-house spectral library [29] into a single .msp file for further mass spectral matching, using the dot product (DP) scoring method included in the MS-DIAL software. 

For both DDA and DIA data analyses, MS-DIAL parameters were set as follows: MS1, tolerance, 0.01 Da; MS2 tolerance, 0.025 Da (0.5 Da for ion trap detection); retention time begin, 0 min; retention time end, 100 min; minimum peak height, 1000; mass slice width, 0.05 Da; smoothing level, 3 scans; minimum peak width, 5 scans; sigma window value, 0.5. 

## 3. Results and Discussion

Our first objective was to develop LC-MS/MS metabolomics workflows on an Orbitrap Fusion^TM^ Tribrid^TM^ instrument thanks to concomitant acquisitions of a MS profile for metabolite detection and quantification, and MS/MS spectra for metabolite identification. We especially aimed at exploiting the versatility of this mass spectrometer, comprising quadrupole, linear ion trap and Orbitrap mass analyzers, in order to conciliate multiple modes of analysis and experimental flexibility. In this context, a set of 47 standard molecules was first used to develop and optimize DDA and DIA (SWATH) acquisition methods, involving either or both non-resonant higher energy collision-induced dissociation (HCD), or ion trap resonant collision-induced dissociation (CID) conditions. 

The 47 chemicals comprising this test set were those already used in a previous study [6] and well ionized in ESI+ mode while exhibiting large chemical, mass and retention time diversity when analyzed on a C_18_ column (Supplementary Material, Table S1). As we observed in a previous study [29], such a C_18_ column is not the most suited one to separate metabolite isomers, but is the most demanding in terms of MS-MS/MS acquisition speed (due to the narrow chromatographic peaks). Therefore, our test set was only made of non-isomeric chemicals, since isomer distinction was not at the center of the present study. Performances of the developed methods were then evaluated and compared using NIST SRM-1950 human plasma as a representative biofluid and considering another set of 72 endogenous metabolites annotated in this matrix (Supplementary Material, Table S5) [29]. 

These developments have been voluntarily carried out on a limited but well characterized set of exogenous and endogenous molecules, to allow further manual data analysis so as to avoid any potential bias induced by processing software tools. A significant part of the data treatment was manually performed while the evaluation of the quality of MS/MS data collected was achieved using the MS-DIAL software.

### 3.1. Implementation of a First “HCD-only” DDA Acquisition Protocol on Authentic Standards as a First Step toward the Collection of Meaningful MS/MS Data

The set of 47 authentic standard molecules was first used as a well known and calibrated mixture in order to optimize experimental conditions (e.g., precursor isolation filters and collision energy applied) and preliminarily evaluate the relevance of a DDA method. Prior to MS detection, metabolite separation was achieved on a C_18_ UHPLC column. Under these conditions, chromatographic peaks are typically less than 10 s wide. In order not to downgrade the quality of MS1 quantification, sufficient data points (at least 8–10) should be obtained per chromatographic peak. Resolutions of the MS1 survey and MS/MS scans were thus kept at 240,000 and 30,000 (both at *m/z* 200, FWHM), respectively. In order to resolve co-selected or co-occurring isobaric precursor or fragment ions, which is a common phenomenon in metabolomics [14], and facilitate spectra identification, Orbitrap resolution was set at the highest value still compatible with the duty cycle. Acquisition of MS data was performed every 10 MS/MS spectra (i.e., a so-called top-10 method) to maintain a total cycle time below 1 s. For comparison, a previously reported and carefully optimized top-5 protocol implemented on an Orbitrap Velos instrument involved mass resolutions set at 30,000 and 7500 in the MS and MS/MS modes, respectively [17]. This highlights the substantial improvement in scan speed (or resolution) of the Orbitrap Fusion^TM^ compared to previous generations of instrument, essentially due to the presence of a high-field Orbitrap mass analyzer.

**Figure 1:**
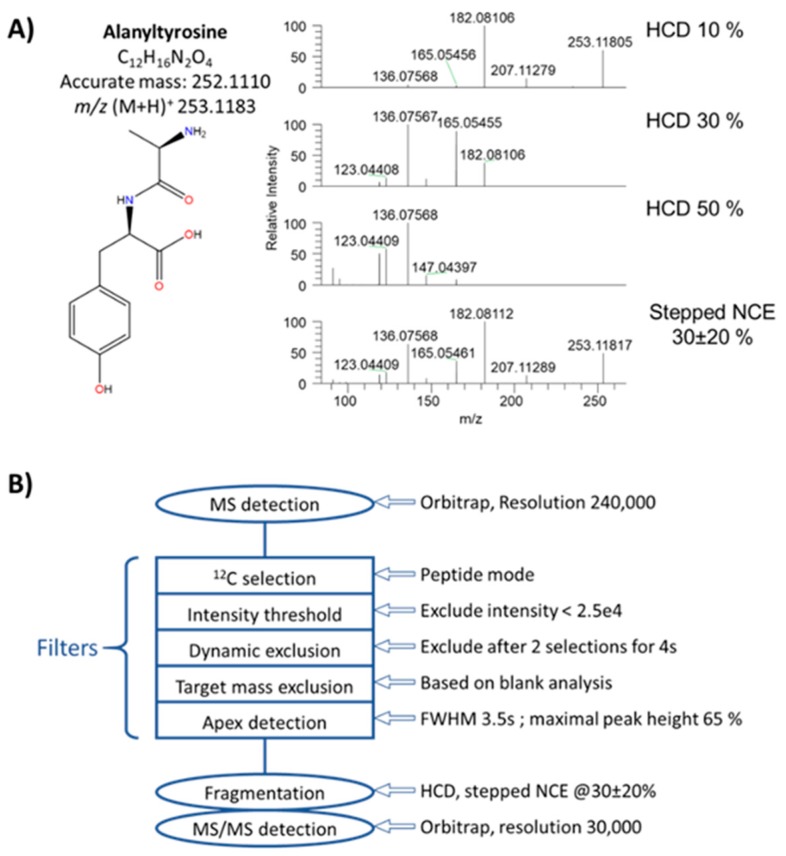
Implementation of an “HCD-only” DDA acquisition workflow. (**A**) MS/MS spectra of alanyltyrosine obtained upon HCD activation at 10%, 30% and 50%, and stepped HCD at 30% ± 20%. (**B**) Schematic overview of the “HCD-only” DDA method (More detailed information regarding experimental conditions and filters is given in Supplementary Material, Table S2).

Efficient DDA methods should enable the acquisition of high-quality and higher purity MS/MS spectra from metabolites of interest present in biological media (see the Supporting Information for information about method development). In this DDA method, non-resonant collision-induced dissociation mode (HCD) was chosen as unique mode of fragmentation. In sharp contrast with proteomics where a single normalized collision energy (NCE) of ~30% is suitable for almost all peptides [32,33], metabolite fragmentation is strongly NCE-dependent and thus no single collision energy can be applied to fit all metabolites [17]. Optimizing applied NCE therefore represents a prerequisite to efficient DDA acquisition protocol in metabolomics. Here, optimal NCE was empirically defined as yielding an MS/MS spectrum with precursor ion at a relative intensity of 15–45% of the base peak. These acquisition conditions often proved to yield the most informative MS/MS spectra, while maintaining the precursor ion detectable. This was also a mean to comparatively evaluate the NCE required to reach similar fragmentation level of given precursor ions. The 47 studied compounds have optimal NCEs varying from 10% to 100% (27% on average, Supplementary Material, Table S1), while ~500 additional compounds from our chemical database and detectable in the positive ion mode overall show optimal NCEs in the 10–50% range (~40% on average, Supplementary Material, Figure S4) [29]. Employing the stepped collision energy option represented a viable way to obtain information-rich MS/MS spectra by combining three different NCEs in a single fragmentation spectrum. A good compromise seems to set stepped NCE at 30 ± 20% (i.e., 10%, 30%, and 50%) to cover most of the compounds presented in Table S1 (Supplementary Material). During such experiments with stepped NCE, the precursor ion is fragmented at 10%, 30%, and 50% NCE, and the fragments from these three energies are combined and detected simultaneously. Under these conditions a single spectrum is obtained. As a representative example, fragmentation of alanyl-tyrosine is shown in Figure 1A with NCEs of 10%, 30%, and 50%, and with a stepped NCE combining those 3 values. Figure 1B shows the whole arrangement of filters, fragmentation and acquisition conditions. 

Before working on the Orbitrap Fusion^TM^, HCD spectra were routinely acquired in our laboratory on a Q-Exactive instrument starting from *m/z* 50 whatever the mass of the precursor ion, so as to highlight potential low-mass diagnostic fragment ions. Surprisingly, when translated to the Orbitrap Fusion^TM^, such experimental conditions led to poor-quality HCD spectra for precursor ions with *m/z* values above ~250 (Figures S1–S3). This observation prompted us to investigate this aspect further by studying the effect of the starting mass on the HCD spectra. Figures S1–S3 display data obtained for 3 representative metabolites, and illustrate the poor detectability of fragment ions in the higher mass range (i.e., exceeding more than ~5 times the lower mass of the selected fragment ion window), due to probable inefficient transmission of the whole ion range. To avoid any detrimental effects when acquiring MS/MS spectra with DDA or DIA workflows, the lower scan limit has been fixed at *m/z* 85. 

**Figure 2:**
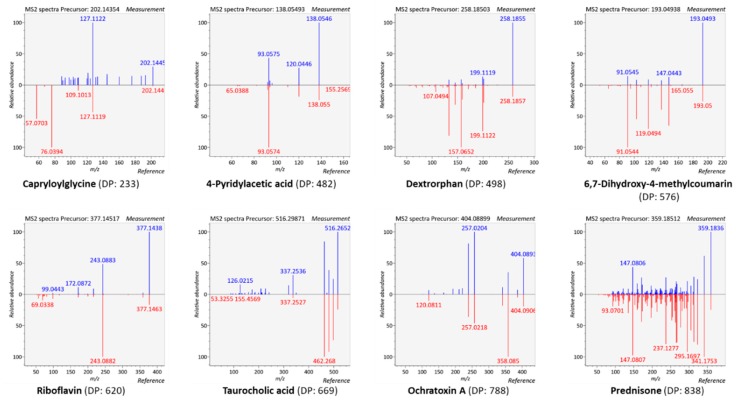
Head-to-tail comparison of evaluated versus reference MS/MS spectra. Evaluated MS/MS spectra were obtained using the HCD-only DDA acquisition workflow.

The pool of 47 molecules was preliminarily analyzed at 100 ng/mL in triplicate by the implemented DDA method with a stepped NCE set at 30% ± 20%. At this rather high concentration, 43/47 compounds were detected in the MS1 survey scan. Limits of detection of 3’-dephosphocoenzyme A, 6-hydroxymelatonin, p-coumaric acid, and dodecanedioic acid were much higher than 100 ng/mL under the conditions used here, thus precluding their efficient detection (Supplementary Material, Table S1). All 43 compounds were efficiently fragmented in each of the three replicates, except ochratoxin A for which no MS/MS spectra were obtained in one replicate. More than 88% of these acquisitions returned a minimum of two MS/MS spectra per replicate for each compound, which highlights data consistency across replicate experiments. Since precursor ion selection occurs in a stochastic fashion within DDA workflows, such a good reproducibility is particularly remarkable. DP scoring was used to evaluate spectral similarity under these DDA conditions. DP scores ranged from 296 to 999 (mean value: 692; median value: 699), with 91% of metabolites having DP scores above 500 (Supplementary Material, Table S7). To better assess and visualize how spectral quality translates into DP score, Figure 2 presents eight MS/MS spectra with variable DP scores. The lowest DP score of 233 was obtained for a rather noisy spectrum of capryloylglycine, but this was the only score below 480. Then comes 4-pyridylacetic acid with a DP of 482 but with a good match between evaluated and reference spectra. Thus, despite fluctuations in the relative intensity of some fragment ions can be observed, a DP score > ~500 seems sufficient to retain high confidence in metabolite identification (Figure 2). Our results are in good agreement with those of Blazenovic et al. who found that a dot product score threshold of 400 gave the best results on a training dataset of more than 300 MS/MS spectra, within the frame of the Critical Assessment of Small Molecule Identification (CASMI) contest [34]. Each 10-fold dilution of test mix concentration (from 100 to 10 and then 1 ng/mL) resulted as expected in a 15–20% drop of the MS/MS triggering efficiency. The global analysis of the recorded data demonstrates the overall efficiency of this primary DDA workflow that sets a solid basis for further methodological developments.

### 3.2. Collecting Meaningful MS/MS Data for Knowns and Unknowns using a DDA Workflow with Parallelized HCD and CID Fragmentations

In untargeted metabolomics, two main categories of compounds can be considered. The first group consists in known metabolites for which a standard compound is available. These metabolites are primarily identified based on their accurate mass and retention time, and then by comparison of their MS/MS spectrum with that of an authentic standard included in an in-house generated reference spectral library. The second group includes “unknown” metabolites that do not find any equivalent in the reference spectral libraries. Those two groups of metabolites can be handled differently when acquiring MS/MS data. In the first case, MS/MS acquisition is “only” to confirm the identity of an annotated metabolite by matching experimental and reference MS/MS spectra acquired under similar fragmentation conditions. Acquisition of MS/MS data at high resolution/high mass accuracy is not compulsory in this particular case. In contrast, for the second category of metabolites, the most complete MS/MS data and accurately measured masses are required to validate a putative annotation or to support a structure proposal based on observed fragment ions. 

**Figure 3:**
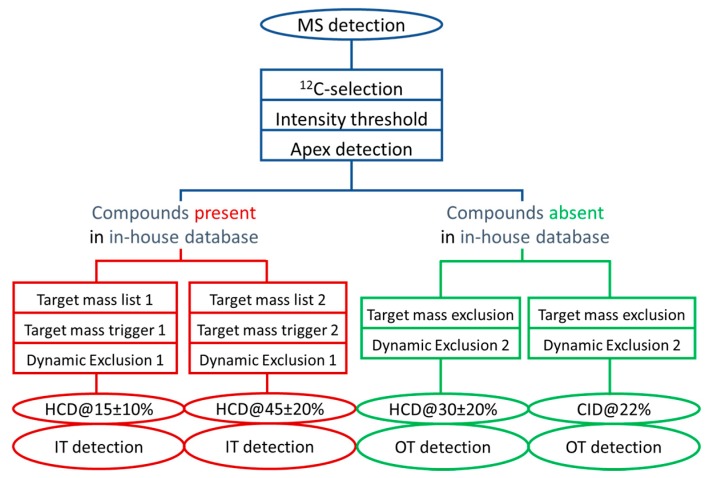
Schematic overview of the DDA method to collect meaningful MS/MS data for knowns and unknowns. See Table S3 for detailed information about the acquisition conditions. IT: Ion Trap, OT: Orbitrap.

Thus, we designed another DDA method whose decision tree is displayed in Figure 3. This second DDA method exploits the versatile capabilities of the Orbitrap Fusion^TM^: (i) metabolite confirmation by parallelizing acquisition of MS1 scans at high resolution in the Orbitrap and low resolution HCD-MS/MS data at a faster rate in the ion trap (red part of Figure 3), and (ii) for characterization of unknowns by acquiring complementary HCD and CID spectra at high resolution (green part of Figure 3).

In this method, we made use of the filters already optimized in the first DDA workflow described above (blue boxes in Figure 3). For metabolites present in our internal database, i.e., for which the corresponding standards are available (red part of the decision tree, Figure 3), the main goal was to obtain high quality MS/MS spectra as close as possible to those present in the spectral database, i.e., with fragments and precursor ion intensities around 20%. To better cover the optimal NCEs of the ~500 chemical compounds included in our chemical database and detectable in the positive ion mode (Supplementary Material, Figure S4) [29], two distinct stepped NCEs of 15% ± 10% and 45% ± 20% were applied for improved reliability of the annotated metabolites identification. For metabolites absent of our chemical library (green part of the decision tree, Figure 3), each corresponding precursor ion was fragmented both under HCD and CID conditions to obtain complementary structural information [17,35], and using compromised stepped NCEs of 30% ± 20% for HCD and 22% for CID. Of note, the multiplexed nature of this multi-event workflow prompted us to lower resolution of MS1 and MS2 events at 120,000 and 15,000, respectively.

We next evaluated the efficiency of this innovative dual DDA method by performing another series of experiments using a methanolic extract obtained from the NIST SRM-1950 plasma spiked with 43 of the above described test mixture of 47 compounds at 10 ng/mL and 100 ng/mL. The NIST plasma is commonly used in our laboratory as a reference sample for method development and data normalization. Up to 72 distinct endogenous metabolites can be routinely detected using our experimental conditions on a C_18_ column and MS detection in the positive ESI mode (Supplementary Material, Table S5). These compounds would be annotated at levels 1 or 3 (in case of isomers, which cannot be discriminated by their MS/MS spectra) according to the guidelines proposed by the Metabolomics Standards Initiative working group [36]. 

For further method evaluation, a working set of 115 molecules was first designed from those 72 endogenous metabolites plus the 43 authentic standards that have been previously used for method development and that demonstrated limits of detection below 100 ng/mL. Then, this working set was randomly split into two subsets of 69 and 46 molecules arbitrarily considered as “present” (i.e., further subjected to HCD MS/MS analyses at low resolution in the ion trap, as displayed in the red part of the decision tree of Figure 3, and referred to as “S1”) or “absent” (i.e., subjected to HCD and CID MS/MS analyses at high resolution in the Orbitrap, as displayed in the green part of the decision tree of Figure 3, and referred to as “S2”) of our chemical library, respectively. The 69 metabolites belonging to the S1 subset have been distributed into two target mass lists depending on their optimal NCE (Figure 3), and were also used to generate an exclusion list for the unknown screening approach. 

**Figure 4:**
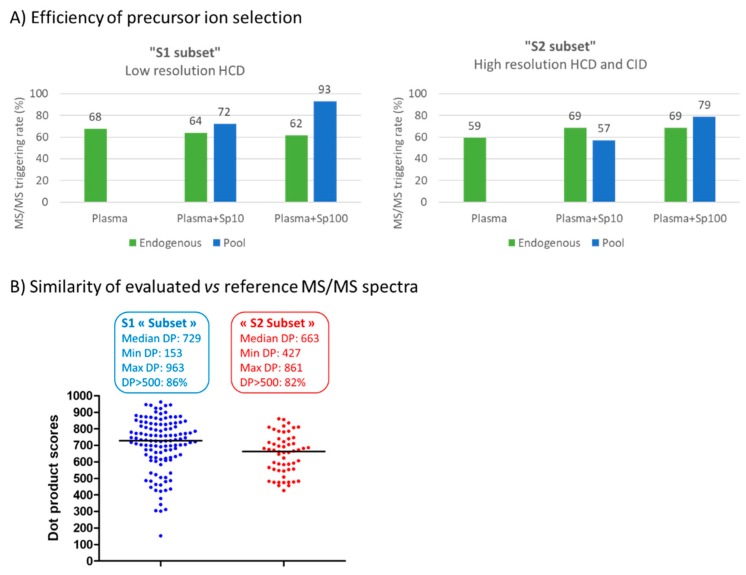
Performance characteristics of the low- and high-mass resolution DDA acquisition workflows on S1 and S2 subsets, respectively. (**A**) Efficiency of precursor ion selection and (**B**) distribution of dot product scores for S1 and S2 subsets. Plasma + Sp10 or Plasma + Sp100 correspond to plasma extracts spiked at 10 and 100 ng/mL, respectively.

Despite 95% of the 115 molecules could be detected at either spiking level, selection of precursor ions was on average ~65% effective, while culminating at more than 80% at the highest spiked concentration for both subsets of compounds (Figure 4A). MS/MS triggering rate also proved similar for compounds subjected to MS/MS experiments at low or high mass resolution. Between 80% and 90% of the molecules spiked at 100 ng/mL were subjected to MS/MS whatever the acquisition workflow. More surprisingly was the fact that only ~65% of the 72 endogenous metabolites triggered MS/MS, whether acquisition was performed under high or low-resolution conditions. We observed that the median retention times of selected and unselected precursor ions were rather similar (1.12 min and 0.92 min, respectively), while their median intensities were significantly different (1.09 × 10^6^ vs. 1.82 × 10^5^, respectively). Therefore, it seems that the less abundant of these endogenous compounds eluting close to the column dead volume were disadvantaged and thus untriggered due to limited acquisition rate of the mass spectrometer especially marked in this retention time region where many compounds coelute, thus leading to dramatic ion suppression effects and competition toward selection for MS/MS. 

Overall, MS/MS spectra obtained at low and high resolution were of high quality and proved comparable to the reference ones, with more than 80% of spectra characterized by a DP score > 500 (Figure 4B). Even though a single non-optimal NCE was used to fragment unknowns (i.e., 30% ± 20%) while maintaining a suitable cycle time, relevant high-resolution mass spectra were still recorded. More specifically regarding the endogenous metabolites monitored in the NIST plasma and belonging to the S2 group, valuable MS/MS spectra were obtained for 15/19 plasma metabolites, i.e., 78% of precursor ions triggering MS/MS acquisition. Manual inspection of the data revealed that this method provided both relevant HCD and CID MS/MS spectra for 10 metabolites, while HCD or CID spectra were retrieved for three and two compounds, respectively.

Altogether, these results emphasize the ability of the Orbitrap Fusion^TM^ to perform high quality DDA for metabolomics. Further software improvements, as those implemented in the AcquireX data dependent software suite (ThermoFisher Scientific), would be useful to improve the presented workflow, for instance regarding automatic exclusion of background signals or for implementing iterative DDA acquisitions in which fragmented metabolites are automatically included in an exclusion list to allow further acquisition of MS/MS data of minor metabolites. 

As shown in this paper, applying different MS/MS acquisition parameters based on predefined exclusion or inclusion lists for proper selection of precursor ions can improve the number of metabolites with high-quality MS/MS spectra. Even exquisite optimization of such gas-phase fractionation procedure on fast-acquiring Q-TOFs, for instance with the joint use of several methods with different mass ranges for selection of precursor ions [37], would not return useful MS/MS spectra for all the metabolites especially those presenting low intensity signals. Thus, we next investigated whether the implementation of DIA workflows might encompass those intrinsic limitations of DDA.

### 3.3. Development of a DIA Acquisition Workflow

For comparison purposes with the DDA method, we also implemented a DIA workflow for untargeted metabolomics applications. Such an approach has the benefit that any detected ion will trigger fragmentation (even those below the selection threshold for DDA) and thus its MS/MS mass spectrum will be collected. The method was built on a full scan MS event followed by a SWATH-like fragmentation, typically using consecutive precursor ion isolation windows with variable sizes (often 20–50 Da) to cover the mass range of interest. To guarantee sufficient data collection rate and chromatographic definition, a fixed number of 10 isolation windows was used and resulting MS/MS spectra were collected in the Orbitrap at a 15,000 resolution, while MS acquisition was performed at 120,000 (both at *m/z* 200, FWHM). In order to reduce the complexity of MS/MS spectra and the width of isolation windows, the studied mass range was limited to 100–600 Da which still well covers the panel of low-mass range metabolites detected in plasma samples (Supplementary Material, Figure S6, lipid species were not considered). The width of the isolation window drastically influences the number of precursor ion peaks isolated and simultaneously fragmented, and thus the complexity of the generated MS/MS spectrum. Therefore, we incrementally devised three distinct DIA workflows involving various isolation windows to obtain the more valuable data. During DIA method development, manual assessment of mass spectral data quality was first performed in a targeted way by specifically looking at the presence of three fragment ions shared with the corresponding spectra from our in-house database. This was also a first step toward the development of a targeted monitoring/quantification DIA-based approach (Section 3.4.2). Then, MS-DIAL software was used to evaluate the consistency of collected MS/MS spectra.

The first one made use of the DIA module included in the Orbitrap Fusion^TM^ software, which automatically defines the number of windows (of equal sizes) according to the mass range studied (10 non-overlapping windows of 50 Da in this case) using a single fixed NCE (set at 30%). When evaluated on the pool of 43 compounds, this approach returned relevant MS/MS spectra for 56.8% of the compounds. When using the “Target MS^2^” acquisition mode of the Orbitrap Fusion software, it became possible to set a stepped NCE of 30% ± 20% and define overlapping 52-Da wide isolation windows. Under these conditions, compound selection and fragmentation were improved, especially at the borders of the isolation windows, thus raising the number of correctly detected compounds to 77.3%. Last, we devised a third acquisition scheme using variable but overlapping precursor isolation window widths (Supplementary Material, Table S4) [38], to partition the precursor ion density observed in plasma (Supplementary Material, Figure S6) almost equally across the whole mass range of interest. Under these conditions, up to 90% of the targeted metabolites were efficiently detected in the NIST plasma extract by their fragment ions (Supplementary Material, Table S1), which clearly underlines the advantages of using variable isolation windows. We next performed mass spectral deconvolution as well as similarity matching within the MS-DIAL software (see below), to more objectively compare the data with those from the above described DDA workflow.

### 3.4. Performance Evaluation of DDA and DIA Strategies

If DDA and DIA acquisition modes can bring valuable information, they both have their own specific drawbacks that are important to know before choosing the best methods toward the objectives of the study. The two strategies were compared on their ability to produce relevant MS/MS information and for quantitative performance characteristics. 

#### 3.4.1. Generation of Meaningful MS/MS Spectra to Confirm the Annotation of Plasma Metabolites 

We next compared DDA and DIA approaches through their respective ability to detect the above-described 72 endogenous metabolites by analyzing in triplicate two independent metabolic extracts of NIST plasma. The methods were qualitatively compared first by manual evaluation and then more thoroughly through DP scores. 

**Figure 5:**
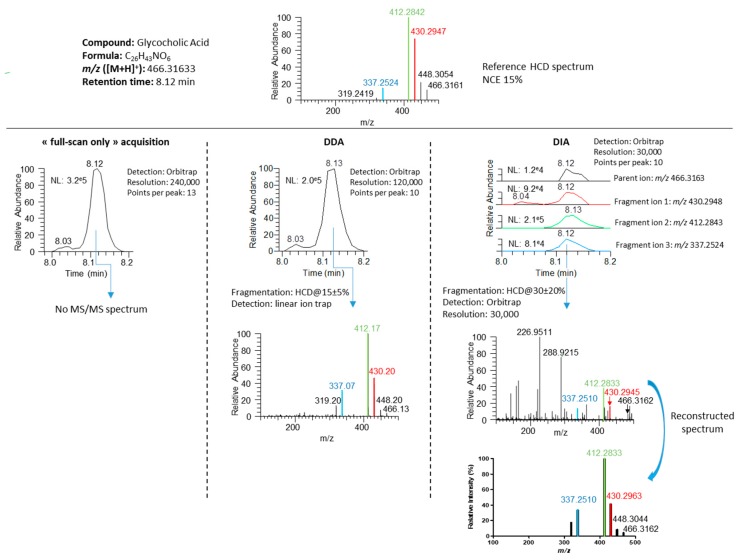
MS and MS/MS data of glycocholic acid obtained with full-scan only, DDA and DIA workflows. The reference MS/MS spectrum was acquired at an NCE of 15% while DDA and DIA acquisitions used NCEs of 15% ± 10% and 30% ± 20%, respectively. DIA MS/MS spectrum of glycocholic acid was manually reconstructed from relative fragment ion intensities.

The DIA workflow provided comprehensive fragmentation of the 72 molecules, whereas only 61% of them were efficiently selected and fragmented by DDA. On average, 2.5 and 11.1 MS/MS spectra per compound were obtained with DDA and DIA approaches, respectively. In both cases, ~90% of the generated spectra proved of sufficient quality to enable manual metabolite confirmation while more than 10 data points/peak also yielded well defined chromatographic peaks for further quantification purposes. Figure 5 shows the data obtained for glycocholic acid as a representative example. These data demonstrate good comparability of MS/MS spectra obtained under DDA and DIA conditions both in terms of presence and relative intensity of the most intense fragment ions (despite the use of distinct fragmentation conditions), and illustrate the higher purity of MS/MS spectra acquired under DDA conditions (Figure 5).

**Figure 6:**
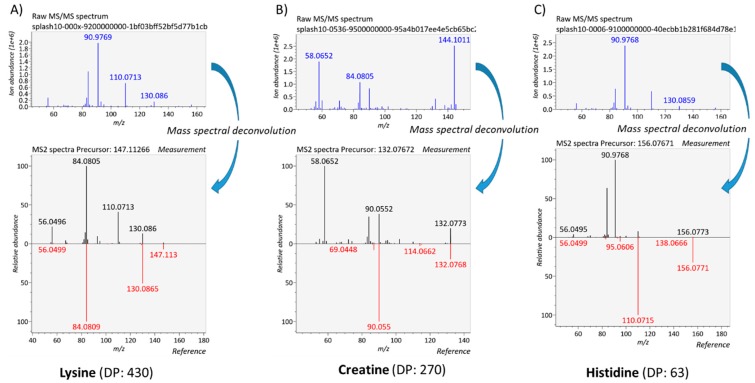
Deconvolution examples of DIA data. (**A**) Lysine with a good DP score, (**B**) creatine whose measured spectrum is contaminated by significant fragment ions arising from co-eluting and co-selected precursor ions but removed by an efficient deconvolution, and (**C**) histidine displaying a very low DP score due to the high abundance of fragment ions arising from other co-fragmented precursor ions.

Table S8 (Supplementary Material) compares the DP scores obtained for 34 metabolites consistently observed for the 6 replicates (two distinct plasma extractions, three technical replicates each) using both DDA and DIA workflows. DP scores returned by both approaches proved consistent between the two extractions. As expected, overall DIA DP scores proved significantly lower with median DP scores of 745 and 628 for DDA and DIA, respectively (Supplementary Material, Table S8). DIA is also characterized by 3 times more metabolites with DP scores below 500 (22 vs. 7). Unsurprisingly, DP scores from DIA measurements seem highly influenced by the quality of mass spectral deconvolution which in turn depends on initial signal intensity and spectral complexity (Figure 6). Even though the DIA can notably benefit from improvements in the deconvolution process, the present results tend to confirm the great potential of such an approach for untargeted metabolomics, with numerous mass spectra exhibiting a rather astonishing spectral quality when compared to reference mass spectra (Supplementary Material, Figure S7).

DDA and DIA acquisition modes are therefore highly complementary and each of them can bring valuable information, depending on the required information. DDA can be used to generate high-quality mass spectra to confirm metabolite annotation or to feed a reference library gathering spectra from known compounds as well as endogenous compounds but still structurally unnamed. DIA approach might be the best choice for targeted or semi-targeted metabolomics projects, within which reference MS/MS spectra are generally available for the metabolites of interest. In this context, particular fragment ions can be targeted to confirm annotations, while fragment ion chromatograms can also be extracted for (semi)quantification purposes despite the lack of direct precursor-fragment link. Since all metabolites are fragmented in such a DIA workflow, all detected metabolites can be basically monitored. 

#### 3.4.2. Quantification of Human Plasma Metabolites from DDA and DIA Modes

One of the main driving forces in metabolomics, following metabolite identification, is the sensitive and robust metabolite quantification. We next intended to compare the quality of relative quantification from DDA and DIA methods by comparison to the reference MS1-only LC-HRMS approach. Then, to further mimic the complexity of real-life complex samples, the set of 43 compounds was spiked into two plasma extracts at concentrations ranging from 0.05 ng/mL to 10 ng/mL and each resulting sample was then analyzed in triplicate. Precursor ion-based quantification was performed from MS1 spectra obtained for both three sets of data, while fragment ion-based quantification was only achieved using DIA data. In the latter case, both precursor ion (when still present) and the three most intense fragments (as deduced from reference MS/MS spectra) were considered. To preliminarily compare measurement reproducibility of both workflows, we first evaluated the number of metabolites detected at a given concentration with a coefficient of variation (CV) on triplicate measurements from the two distinct plasma extracts below 30%. 

As shown in Figure S8 (Supplementary Material), the acquisition of MS/MS events, either in DDA or in DIA has no drastic impact on the number of molecules being detected with CV < 30% at each concentration level. Importantly, this demonstrates that adding MS/MS events does not downgrade the quality and robustness of the measurement at the MS1 level, which is consistent with the results recently reported using a fast-acquiring Q-TOF instrument and a similar analytical strategy [25]. Overall, precursor ion-based monitoring from MS1 signals proved of equal sensitivity whatever the acquisition method, while the DIA-MS/MS strategy proved less sensitive with on average ~5 times less compounds detected. This might be related to incomplete efficiency of ion transmission and ion fragmentation using non-optimal NCE conditions. This is in sharp contrast with targeted parallel reaction monitoring (PRM)-based metabolomics approaches where NCE is carefully optimized in a compound-specific manner to reach exquisite sensitivity [39]. Nevertheless, at the highest concentrations, the average measurement reproducibility proved comparable using MS1 and MS/MS ion intensities.

**Figure 7:**
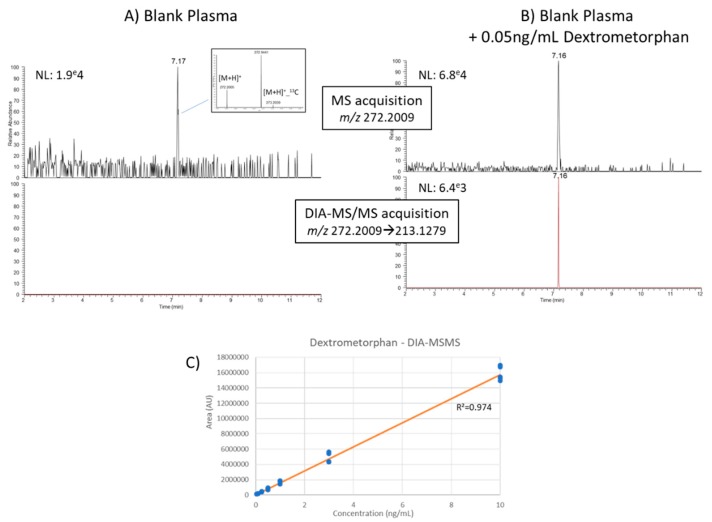
Example of Dextrometorphan. (**A**) A metabolite feature probably misidentified in blank plasma as dextromethorphan by the full-scan MS method and not confirmed by the DIA-MS/MS approach, (**B**) similar data as in (**A**) but after spiking 0.05 ng/mL of pure dextromethorphan in plasma. (**C**) calibration curve of dextromethorphan in human plasma as analyzed by DIA-MS/MS.

Last, we studied in more detail the performances of the four workflows for the relative quantification of nine spike-in compounds with estimated limits of detection in the 0.05–0.25 ng/mL range (Supplementary Material, Table S9). Overall, MS-only and DDA-MS workflows shared similar detection sensitivities, while quantification based on DIA-MS (MS1 trace of DIA data) and DIA-MS/MS data proved ~2- and ~5-times less sensitive. Concentration-response relationship was demonstrated as linear over at least two orders of magnitude whatever the acquisition workflow with average coefficients of determination R^2^ > 0.97 (Supplementary Material, Table S9). Similarly, all four workflows demonstrated similar good quantification accuracies as measured at 0.25 and 3 ng/mL concentration levels (with accuracies ranging from 81–108%, and 94–104%, respectively). Although significantly less sensitive, the DIA-MS/MS approach enables simultaneous metabolite structural confirmation and quantification. For example, DIA-MS/MS data proved useful to highlight potential misannotation of dextromethorphan (cough suppressant) in blank human plasma after analysis by the full-scan only method, while also enabling its quantification in the 0.05–10 ng/mL range (Figure 7).

## 4. Conclusions

Performance of the Orbitrap Fusion^TM^ instrument, equipped with a compact high-field Orbitrap analyzer, was studied for the efficient MS/MS data collection in an untargeted metabolomics context. Our data indicated that both DDA and SWATH-type DIA approaches are highly valuable and proved complementary, DDA not surprisingly being more suited for acquiring high-quality and higher-purity spectra. The unprecedented versatility of the Orbitrap Fusion^TM^ enables parallelized DDA acquisition of both high resolution and low resolution spectra as well as concomitant collection of HCD and CID data, which is particularly relevant for identification of unknowns. Interestingly, comparative quantitative evaluation of those multi-event approaches revealed that the acquisition of MS/MS spectra, either in DDA or in DIA, does not downgrade the quality of the quantification at the MS1 level. While significantly less sensitive (~five times), fragment ion-based quantification from DIA-MS/MS data enables simultaneous metabolite structural confirmation and quantification of known metabolites. Most of the methodological developments reported here on the Orbitrap Fusion and drawn conclusions are also relevant for other Orbitrap-based platforms, such as Q-Exactive or LTQ-Orbitrap instruments. Both DDA and DIA approaches for metabolomics would greatly benefit from improvements in data acquisition speed on Orbitrap mass analyzers as well as in NCE management to better cover the wide heterogeneity of metabolites in terms of optimal fragmentation conditions. Given the results of this study, we conclude that combining metabolite quantification in the MS1 space and DIA-MS/MS for obtaining structural information of all detected compounds and querying high-quality spectral databases (generated by targeted DDA at multiple NCEs), can have great potential for future biological metabolomics applications.

## Supplementary Materials

The following are available online at https://www.mdpi.com/2218-1989/10/4/158/s1, Figure S1: Comparison of HCD spectra acquired for 4-pyridylacetic acid on the Orbitrap Fusion^TM^ or on a Q-Exactive^TM^ using variable *m/z* windows for fragment ions; Figure S2: Comparison of HCD spectra acquired for propanolol on the Orbitrap Fusion^TM^ or on a Q-Exactive^TM^ using variable *m/z* windows for fragment ions; Figure S3: Comparison of HCD spectra acquired for ochratoxin A on the Orbitrap Fusion^TM^ or on a Q-Exactive^TM^ using variable *m/z* windows for fragment ions; Figure S4: Distribution of optimal collision energy for 482 standard metabolites; Figure S5: Distribution of optimal collision energy for 72 standard metabolites identified in NIST plasma using C_18_ UHPLC system coupled to (ESI+)-MS/MS; Figure S6: Mass distribution of metabolites identified in plasma; Figure S7: Head-to-tail comparison of evaluated versus reference MS/MS spectra. Evaluated MS/MS spectra were obtained using the DIA acquisition workflow; Figure S8: Number of metabolites reproducibly detected (with CV < 30%) using full-scan only (blue), DDA (red), DIA-MS (green) and DIA MS/MS workflows (purple). Each bar is the result of six independent measurements, Text S1. Optimisation of a first “HCD-only” DDA method; Table S1: List of the 47 compounds included in the test sample as analyzed under ESI+ conditions; Table S2: Acquisition parameters of the “reference” DDA workflow; Table S3: Acquisition parameters of the DDA workflow combining low- and high-mass resolution acquisitions; Table S4: Acquisition parameters of the DIA workflow; Table S5: List of the 72 metabolites annotated in plasma NIST in C18 LC condition and ESI+ ionization; Table S6: Example of an exclusion list generated from contaminant background ions; Table S7: Dot product scores obtained for the standard mixture by using the HCD-only DDA workflow (mean values of three replicate measurements); Table S8: Dot product scores obtained by DDA and DIA for a set of 34 metabolites (mean of three replicates per extraction); Table S9: Comparison of performance characteristics of MS-Only, DDA-MS, DIA-MS, and DIA-MS/MS workflows.
