# Supplementary material for: Comparative Evaluation of Data Dependent and Data Independent Acquisition Workflows Implemented on an Orbitrap Fusion for Untargeted Metabolomics
Source: Metabolites. 2020 Apr 18;10(4):158. doi: 10.3390/metabo10040158 (PMC7240956; doi:10.3390/metabo10040158)
Supplement: Supplementary file 1 [file metabolites-10-00158-s001.pdf]

SUPPLEMENTAL INFORMATION

Comparative Evaluation of Data Dependent and Data Independent Acquisition Workflows Implemented on an Orbitrap Fusion for Untargeted Metabolomics

Pierre BARBIER SAINT HILAIRE[1,#], Kathleen ROUSSEAU[1,#], Alexandre SEYER[2], Sylvain DECHAUMET[2], Annelaure DAMONT[1], Christophe JUNOT[1], François FENAILLE*[,1]

[1] Université Paris-Saclay, CEA, INRAE, Département Médicaments et Technologies pour la Santé (DMTS), MetaboHUB, F-91191 Gif sur Yvette, France.

[2] MedDay Pharmaceuticals SA, 24 Rue de la Pépinière, F-75008 Paris, France.

[*] E-mail: francois.fenaille@cea.fr

Text S1. Optimisation of a first "HCD-only" DDA method

We first optimized precursor selection filters such as intensity threshold, target mass exclusion, dynamic exclusion, apex detection, and monoisotopic precursor selection (Table S2, Supporting Information). In particular, special attention was paid to excluding frequently observed irrelevant background ions ("target mass exclusion" filter), not to waste time collecting MS/MS data on meaningless signals originating from various sources of contaminants (e.g., mobile phases, solvents, formic acid clusters). A solvent blank sample was analyzed prior to each acquisition batch to (manually) generate an exclusion list from detected background signals present at rather high intensities above 1e5 (to avoid exclusion of any minor metabolite signals resulting from slight but significant sample-to-blank memory or carry-over effects) with an *m/z* tolerance of 5 ppm. As mobile phase composition changes according to the gradient, contaminant detection was accomplished throughout the whole chromatographic run. An example of such an exclusion list is given in Table S6 (Supporting Information), including more than 50 distinct recurrently observed rather abundant contaminant ions. Target ions (produced from relevant metabolites) already selected for MS/MS were dynamically excluded after two selections for 4 seconds ("dynamic exclusion" filter), which corresponds to the typical chromatographic peak full width at half maximum. Similarly, the "apex detection" filter proved efficient for selecting an *m/z* signal at its highest intensity. Last, the best choice for the "monoisotopic precursor selection" (MIPS) filter to avoid selection of ^{13}C-isotopes was rather unexpectedly demonstrated to be the "peptides" setting and not the "small molecules" one (Table S2, Supporting Information). Of note, this filter should remain unselected when working on chemical compounds exhibiting particular isotopic patterns such as those incorporating chlorine or bromine atoms.

Table S1. List of the 47 compounds included in the test sample as analyzed under ESI+ conditions.
Under our experimental conditions, a few compounds did not yield $[M+H]^+$ as most intense species but rather fragment ions resulting from in-source loss(es) of water.

**Compound**	**Composition**	**[M+H]+**	**Retention time (min)**	**Approximative optimal NCE (%)**	**3 Most Intense Fragment ions**	**Estimated limit of detection in solvent (ng/mL)**
2-Aminophenol	C6H7NO	110.0600	1.12	40	92.0496/65.0387/82.0649	1
Nicotinic acid	C6H5NO2	124.0393	0.93	100	96.0445/112.0392/80.0497	1
Isoleucine	C6H13NO2	132.1019	1.26	15	86.0966	0.1
4-Pyridylacetic acid	C7H7NO2	138.0550	0.83	80	93.0575/120.0445/94.0652	0.1
Triethanolamine	C6H15NO3	150.1125	0.75	40	132.1019/88.0758/114.0914	0.1
p-Coumaric acid	C9H8O3	165.0546	5.77	10	147.0441/164.9300/141.9789	>100
4-Methylumbelliferone	C10H8O3	177.0546	6.6	50	103.0544/121.0649/91.0544	10
6,7-Dihydroxy-4-methylcoumarin	C10H8O4	193.0495	5.66	40	147.044/119.0492/103.0544	1
Caprylolyglycine	C10H19NO3	202.1438	7.78	15	184.1332/127.1118/109.1013	10
Pantothenic acid	C9H17NO5	220.11795	2.42	20	90.0551/202.1074/184.0968	1
Flavone	C15H10O2	223.0754	9.35	80	121.0286/103.0547/129.0338	1
Dodecanedioic acid	C12H22O4	231.1591	8.34	15	213.1482/167.1429/149.1323	>100
6-Hydroxymelatonin	C13H16N2O3	249.1234	5.4	10	232.0969/190.0862/158.0602	>100
Ala-Tyr	C12H16N2O4	253.1183	1.68	10	182.0811/136.0757/165.0546	0.1
Dextrorphan	C17H23NO	258.1852	5.89	65	199.1124/133.0653/201.1277	0.1
(±)-Propranolol	C16H21NO2	260.1645	7.1	30	116.1071/183.0803/155.0855	0.1
Formononetin	C16H12O4	269.0808	8.33	50	254.0570/213.0913/253.0499	0.1
Dextromethorphan	C18H25NO	272.2009	7.2	50	215.1433/213.1279/147.0807	0.1
19-Nortestosterone	C18H26O2	275.2006	8.65	40	109.065/257.1901/105.0701	1
Testosterone	C19H28O2	289.2162	9.01	30	109.0649/271.2058/253.1953	1
Atropine	C17H23NO3	290.1751	5.6	40	124.1122/93.0701/91.0546	0.1
D-Sphingosine (-H2O)	C18H37NO2 (C18H35NO)	300.2897 (282.2791)	10.63	10	282.2789/211.2057/252.2683	100
(-)-Scopolamine	C17H21NO4	304.1543	5	40	138.0914/103.0546/121.0649	0.1
7a-Hydroxytestosterone	C19H28O3	305.2111	6.86	30	287.2005/269.1900/145.1011	1
Stanozolol	C21H32N2O	329.2587	9.22	80	121.1013/107.0857/105.0701	0.1
21-Deoxycortisol	C21H30O4	347.2217	7.99	30	311.2004/121.0648/147.0805	1
Prednisone	C21H26O5	359.1853	7.24	20	341.1748/147.0806/171.0807	1
Curcumin	C21H20O6	369.1333	9.39	20	177.0545/245.0809/285.1117	1
Cholic acid (-2H2O)	C24H40O5 (C24H36O3)	409.29485 (373.2737)	8.89	20	355.26.27/159.1167/145.1011	10
Finasteride	C23H36N2O2	373.2850	9.26	60	305.259/317.2226/121.1013	0.1
Riboflavin	C17H20N4O6	377.1456	5.19	30	243.0874/172.0869/216.0767	1
trans-Zeatin glucoside	C16H23N5O6	382.1721	2.93	30	220.1191/202.1091/136.0618	0.1
Ochratoxin A	C20H18ClNO6	404.08954	9.39	20	239.0104/257.0209/358.0836	10
Lincomycin	C18H34N2O6S	407.2210	4.88	30	126.1278/359.2177/389.2101	0.1
Folic acid	C19H19N7O6	442.14696	4.64	10	295.0934/176.0566/313.1041	1
Glycodeoxycholate	C26H43NO5	450.3214	9.19	10	414.3001/432.31/339.2679	10
Psychosine	C24H47NO7	462.3425	9.84	20	444.3321/282.2792/264.2686	10
Glycocholic acid	C26H43NO6	466.3163	8.12	10	430.2948/412.2843/337.2524	1
Deoxycorticosterone 21-glucoside	C27H40O8	493.2796	7.57	20	331.2264/313.2162/145.0496	1
Taurodeoxycholic acid	C26H45NO6S	500.3040	8.28	10	464.2824/126.0220/482.2929	10
Taurocholic acid (-H2O)	C26H45NO7S (C26H43NO6S)	516.2990 (498.2867)	7.46	10	462.2673/480.2778/337.2522	10
D-Pantethine	C22H42N4O8S2	555.2517	5.55	10	425.1887/295.1254/147.0587	1
a-Ergocryptine	C32H41N5O5	576.3181	7.59	30	223.1233/268.1447/558.3087	1
Apigenin 7-O-neohesperidoside	C27H30O14	579.1708	6.18	15	271.0598/433.1129/129.0547	1

Naringin	C27H32O14	581.18648	6.24	10	273.0757/419.1331/435.1272	10
Rutin	C27H30O16	611.1607	5.73	10	465.1026/303.0498/449.1078	10
3'-Dephosphocoenzyme A	C21H35N7O13P2S	688.1562	2.05	20	428.036/348.0699/261.1264	>100

Table S2. Acquisition parameters of the “reference” DDA workflow

**MS1 acquisition**	
Scan range	*m/z* 85-1000
Resolution	240,000 at *m/z* 200 (FWHM)
Maximum injection time	400 ms
AGC target	5e4
**MS/MS acquisition**	
Activation	HCD
Resolution	30,000 at *m/z* 200 (FWHM)
AGC target	5e4
Maximum injection time	54 ms
Isolation width	0.8 Da
Stepped collision energy	10, 30, 50%
Filters	
Intensity threshold	2.5e4
Exclusion list	Intensity>1e5, 5ppm (see Table S3 as an example)
Dynamic exclusion	4 s, after 2 repetitions in 1s
Apex detection	3.5 s, 65% maximal peak height
Monoisotopic precursor selection (MIPS)	Peptide

Table S3. Acquisition parameters of the DDA workflow combining low- and high-mass resolution acquisitions.

**MS1 acquisition**	
Scan range	*m/z* 85-1000
Resolution	120,000 at *m/z* 200 (FWHM)
AGC target	4e5
**MS/MS acquisition for "present" molecules ("S1 subset")**	
Activation	HCD
Detection	Ion Trap
Resolution	Normal
AGC target	1e4
Maximum injection time	35 ms
Isolation width	0.8 Da
Stepped collision energy	15 ±10% and 45 ±20%
**MS/MS acquisition for "absent" molecules ("S2 subset")**	
Activation	HCD and CID
Detection	Orbitrap
Resolution	15,000 at *m/z* 200 (FWHM)
AGC target	5e4
Maximum injection time	22 ms
Isolation width	0.8 Da
Collision energy	HCD@30 ±20%, CID@22%
Common filters for "S1 and S2 subsets"	
Intensity threshold	2.5e4
Apex detection	3.5 s, 65% maximal peak height
Monoisotopic precursor selection (MIPS)	Peptide
Filters for "S1 subset"	
Targeted Mass	Compounds of interest (m/z +/- 5ppm, RT range)
Targeted Mass trigger	Compounds of interest (m/z +/-5ppm)
Dynamic exclusion	8 s, after 3 repetitions in 3s
Filters for "S2 subset"	
Exclusion list	All compounds from the "S1 subset" and all compounds with an intensity > 1.10^5 in blank samples (5 ppm)
Dynamic exclusion	4 s, after 2 repetitions in 1.5s

Table S4. Acquisition parameters of the DIA workflow

**MS1 acquisition**	
Scan range	*m/z* 85-1000
Resolution	120,000 at *m/z* 200 (FWHM)
Maximum injection time	200 ms
AGC target	5e4
**MS/MS acquisition**	
Activation	HCD
Resolution	15,000 at *m/z* 200 (FWHM)
AGC target	5e4
Maximum injection time	22 ms
Stepped collision energy	30 ±20%
m/z Windows	
1	98 – 132
2	129.5 – 145.5
3	144.5 – 160.5
4	159.5 – 175.5
5	174.5 – 190.5
6	189 – 211
7	209 – 262
8	259 – 301
9	300 – 400
10	399 - 602

Table S5. List of the 72 metabolites annotated in plasma NIST in C18 LC condition and ESI+ ionization

Name	Formula	Mass	Retention Time (min)
Uracil	C4H4N2O2	112.0273	1.06
Creatinine	C4H7N3O	113.0589	0.83
Proline	C5H9NO2	115.0633	0.85
Betaine	C5H11NO2	117.0790	0.82
Valine	C5H11NO2	117.0790	0.93
Threonine / D-allo-Threonine	C4H9NO3	119.0582	0.84
Nicotinamide	C6H6N2O	122.0480	0.99
Pyroglutamic-acid	C5H7NO3	129.0426	1.08
Pipecolinic-acid	C6H11NO2	129.0790	0.94
Creatine	C4H9N3O2	131.0695	0.84
Isoleucine	C6H13NO2	131.0946	1.28
Leucine	C6H13NO2	131.0946	1.38
5-Hydroxyindole	C8H7NO	133.0528	4.92
Hypoxanthine	C5H4N4O	136.0385	0.99
Trigonelline	C7H7NO2	137.0477	0.84
4-Imidazoleacrylic acid	C6H6N2O2	138.0429	0.93
Methylimidazoleacetic-acid	C6H8N2O2	140.0586	0.86
Stachydrine	C7H13NO2	143.0946	0.85
4-Guanidinobutyric-acid	C5H11N3O2	145.0851	0.93
Glutamine	C5H10N2O3	146.0691	0.81
Lysine	C6H14N2O2	146.1055	0.72
L-Glutamic-acid	C5H9NO4	147.0532	0.82
Methionine	C5H11NO2S	149.0511	1.04
Acetaminophen-(4-Acetamidophenol)	C8H9NO2	151.0633	2.88
Histidine	C6H9N3O2	155.0695	0.76
Carnitine	C7H15NO3	161.1052	0.82
1-methyl-guanine / 7-methylguanine	C6H7N5O	165.0651	0.97
Phenylalanine	C9H11NO2	165.0790	2.10
7-Methylxanthine	C6H6N4O2	166.0491	1.45
3-Methylxanthine	C6H6N4O2	166.0491	1.60
1-Methylxanthine	C6H6N4O2	166.0491	1.74
Uric-acid	C5H4N4O3	168.0283	0.94
1-Methylhistidine	C7H11N3O2	169.0851	0.78
Arginine	C6H14N4O2	174.1117	0.77
L-Citrulline	C6H13N3O3	175.0957	0.81
Cotinine	C10H12N2O	176.0950	1.08
Hippuric-acid	C9H9NO3	179.0582	4.93
paraxanthine / Theophylline	C7H8N4O2	180.0647	3.95
Theobromine	C7H8N4O2	180.0647	2.52
Tyrosine	C9H11NO3	181.0739	1.16
3-Amino-3-(4-hydroxyphenyl)propanoic	C9H11NO3	181.0739	1.17
1-Methyluric-acid	C6H6N4O3	182.0440	1.46
4-Pyridoxic-acid	C8H9NO4	183.0532	1.37
N-acetyl-L-glutamine	C7H12N2O4	188.0797	0.94
N6-Acetyl-L-lysine	C8H16N2O3	188.1161	0.90
N6,N6,N6-Trimethyl-L-lysine	C9H20N2O2	188.1525	0.76
Trans-3-Hydroxy-cotinine	C10H12N2O2	192.0899	0.91
(S)-Cotinine-N-oxide	C10H12N2O2	192.0899	1.52
Caffeine	C8H10N4O2	194.0804	4.89
Acetyl-L-carnitin	C9H17NO4	203.1158	1.01
Tryptophan	C11H12N2O2	204.0899	4.24
Panthenol	C9H19NO4	205.1314	2.48

L-Kynurenine	C10H12N2O3	208.0848	2.11
N-alpha-acetyl-L-arginine	C8H16N4O3	216.1222	0.90
Propionylcarnitine	C10H19NO4	217.1314	1.54
Pantothenic-acid	C9H17NO5	219.1107	2.45
(R)-Butyryl-carnitine	C11H21NO4	231.1471	3.48
L-Cystine	C6H12N2O4S2	240.0239	0.84
L-a-Glycerophosphorylcholine	C8H20NO6P	257.1028	0.77
Hexanoylcarnitine	C13H25NO4	259.1784	6.19
Phenylacetyl-L-glutamine	C13H16N2O4	264.1110	5.07
N-Acetyl-L-carnosine	C11H16N4O4	268.1172	0.88
1-Methyladenosine	C11H15N5O4	281.1124	0.92
Octanoylcarnitine	C15H29NO4	287.2097	7.63
5-Deoxy-5-(methylthio)adenosine	C11H15N5O3S	297.0896	4.16
Decanoylcarnitine	C17H33NO4	315.2410	8.79
Acetaminophen-glucuronide	C14H17NO8	327.0954	1.56
Sphingosine-1-phosphate	C18H38NO5P	379.2488	10.24
glycochenodeoxycholic-acid	C26H43NO5	449.3141	9.12
glycodeoxycholate	C26H43NO5	449.3141	9.29
Glycocholic-acid	C26H43NO6	465.3090	8.21
Stercobilin	C33H46N4O6	594.3417	7.16

Table S6. Example of an exclusion list generated from contaminant background ions

m/z	Start time (min)	End time (min)
90.97677	5	20
99.51234	8	14
103.95569	8	14
113.96374	0	14
114.0914	0	14
116.97196	8	14
121.9662	8	20
123.96438	8	20
125.98627	5	8
130.15904	0	14
139.98788	8	14
144.98213	8	20
146.98031	8	20
149.02328	0	14
150.02661	0	5
158.15388	0	5
158.96401	5	20
189.05138	0	8
194.11747	0	14
195.08755	0	5
199.18038	0	14
220.93438	5	14
226.95133	0.8	20
245.11387	0	5
268.24527	0	0.8
274.27385	8	14
279.15883	0	0.8
282.22136	0	14
288.9217	0	14
301.14073	0	5
306.89443	8	14
362.92603	5	20
415.21113	8	14
424.89646	5	14
427.37781	0	5
430.91334	5	20
476.30604	5	8
492.88401	8	14
498.90092	8	20
566.88831	8	20
634.87566	8	20
702.86305	8	20
703.57444	8	14
758.56899	8	14
759.57238	8	14
760.58458	8	14
761.58797	8	14
770.85044	8	20
782.56788	8	20
783.57138	8	14
784.58444	8	14
786.60024	8	14

787.60364	8	14
804.55078	8	14
806.56801	8	14
808.5826	8	14
810.60005	8	14
265.96213	14	20
838.83751	14	20

Table S7. Dot product scores obtained for the standard mixture by using the HCD-only DDA workflow (mean values of three replicate measurements).

**Metabolite**	**Dot product**
2-Aminophenol	999
Nicotinic acid	480
Isoleucine	687
4-Pyridylacetic acid	569
Triethanolamine	645
4-Methylumbelliferone	508
6,7-Dihydroxy-4-methylcoumarin	581
Capryloylglycine	296
Pantothenic acid	752
Flavone	727
Ala-Tyr	810
Dextrorphan	489
(±)-Propranolol	674
Formononetin	495
Dextromethorphan	551
19-Nortestosterone	685
Testosterone	646
Atropine	578
D-Sphingosine	720
(-)-Scopolamine	693
7a-Hydroxytestosterone	676
Stanozolol	939
21-Deoxycortisol	761
Prednisone	839
Curcumin	773
Cholic acid	630
Finasteride	544
Riboflavin	620
trans-Zeatin glucoside	758
Ochratoxin A	779
Lincomycin	805
Folic acid	672
Glycodeoxycholate	729
Psychosine	852
Glycocholic acid	770
Deoxycorticosterone 21-glucoside	804
Taurodeoxycholic acid	733
Taurocholic acid (-H2O)	718
D-Pantethine	617
a-Ergocryptine	791
Apigenin 7-O-neohesperidoside	874
Naringin	699
Rutin	807

Table S8. Dot product scores obtained by DDA and DIA for a set of 34 metabolites (mean of 3 replicates per extraction).

	DDA		DIA	
**Metabolite**	Mean Dot Product Extraction 1	Mean Dot Product Extraction 2	Mean Dot Product Extraction 1	Mean Dot Product Extraction 2
Proline	460	604	626	605
Betaine	750	641	656	657
Pyroglutamic-acid	579	588	465	478
Creatine	807	810	236	247
Leucine/Isoleucine	747	749	621	708
Hypoxanthine	596	615	570	552
Trigonelline	725	742	30	35
Stachydrine	868	870	570	596
Glutamine	741	769	467	467
Lysine	841	852	415	447
Methionine	861	931	585	630
Acetaminophen-(4-Acetamidophenol)	708	714	708	737
Histidine	783	748	51	51
Carnitine	489	495	643	694
Phenylalanine	656	662	534	535
Uric-acid	863	824	765	805
1-Methylhistidine	542	585	133	97
Arginine	778	807	688	685
Cotinine	810	805	831	830
Paraxanthine / Theophylline	755	690	764	763
Theobromine	762	693	947	948
Tyrosine	831	838	709	711
Trans-3-Hydroxy-cotinine	840	789	672	703
Tryptophan	835	841	517	515
Propionylcarnitine	853	848	919	913
Pantothenic-acid	505	479	338	402
(R)-Butyryl-carnitine	598	483	855	689
Hexanoylcarnitine	487	477	741	743
Phenylacetyl-L-glutamine	900	898	898	899
Decanoylcarnitine	566	660	873	870
Acetaminophen-glucuronide	557	590	452	449
Sphingosine-1-phosphate	764	721	193	163
Glycochenodeoxycholic-acid	822	742	865	866
Glycocholic-acid	664	513	431	376

Table S9. Comparison of performance characteristics of MS-Only, DDA-MS, DIA-MS, and DIA-MS/MS workflows

Metabolite	MS-Only					DDA-MS				
	Estimated LOD (ng/mL)[a]	Dynamic range (ng/mL)	r2	Accuracy @0.25 ng/mL	Accuracy @3 ng/mL	Estimated LOD (ng/mL)[a]	Dynamic range (ng/mL)	r2	Accuracy @0.25 ng/mL	Accuracy @3 ng/mL
Scopolamine	0.05	0.05-10	0.99	102	96	0.05	0.05-10	0.99	102	98
Flavone	0.25	0.25-10	0.95	108	93	0.1	0.1-3	0.93	81	102
Formononetin	0.5	0.5-10	0.96	103	85	0.25	0.25-10	0.95	107	86
Finasteride	0.1	0.1-3	0.92	83	104	0.1	0.1-3	0.92	86	103
Propanolol	0.05	0.05-10	0.98	98	99	0.05	0.05-10	0.99	99	99
trans-ZeatinGlucoside	0.25	0.25-10	0.97	103	94	0.25	0.25-10	0.97	105	95
Dextrorphan	0.05	0.05-10	0.98	97	99	0.05	0.05-10	0.98	97	98
Lincomycin	0.25	0.25-10	0.97	105	96	0.05	0.05-10	0.98	96	100
alpha-Ergocryptine	0.5	0.5-10	0.98	103	94	0.25	0.25-10	0.98	100	94

Metabolite	DIA-MS					DIA-MS/MS				
	Estimated LOD (ng/mL)[a]	Dynamic range (ng/mL)	r2	Accuracy @0.25 ng/mL	Accuracy @3 ng/mL	Estimated LOD (ng/mL)[a]	Dynamic range (ng/mL)	r2	Accuracy @0.25 ng/mL	Accuracy @3 ng/mL
Scopolamine	0.05	0.05-10	0.98	100	97	0.25	0.25-10	0.98	102	97
Flavone	0.1	0.1-3	0.92	84	102	0.25	0.25-10	0.96	107	94
Formononetin	3	3-10	NA	NA	NA	3	3-10	NA	NA	NA
Finasteride	0.1	0.1-3	0.92	85	102	0.25	0.25-10	0.96	109	98
Propanolol	0.05	0.05-10	0.98	99	98	0.1	0.1-10	0.98	100	99
trans-ZeatinGlucoside	0.25	0.25-10	0.97	103	94	3	3-10	NA	NA	NA
Dextrorphan	0.05	0.05-10	0.98	98	99	0.1	0.1-10	0.98	101	98
Lincomycin	0.25	0.25-10	0.97	105	95	3	3-10	NA	NA	NA
alpha-Ergocryptine	0.25	0.25-10	0.98	101	95	0.5	0.5-10	0.97	100	99

[a] Determined as the lowest calibration point with a CV<20%

NA: Not Applicable

Highlighted with a yellow background are the accuracies measured at 0.5ng/mL

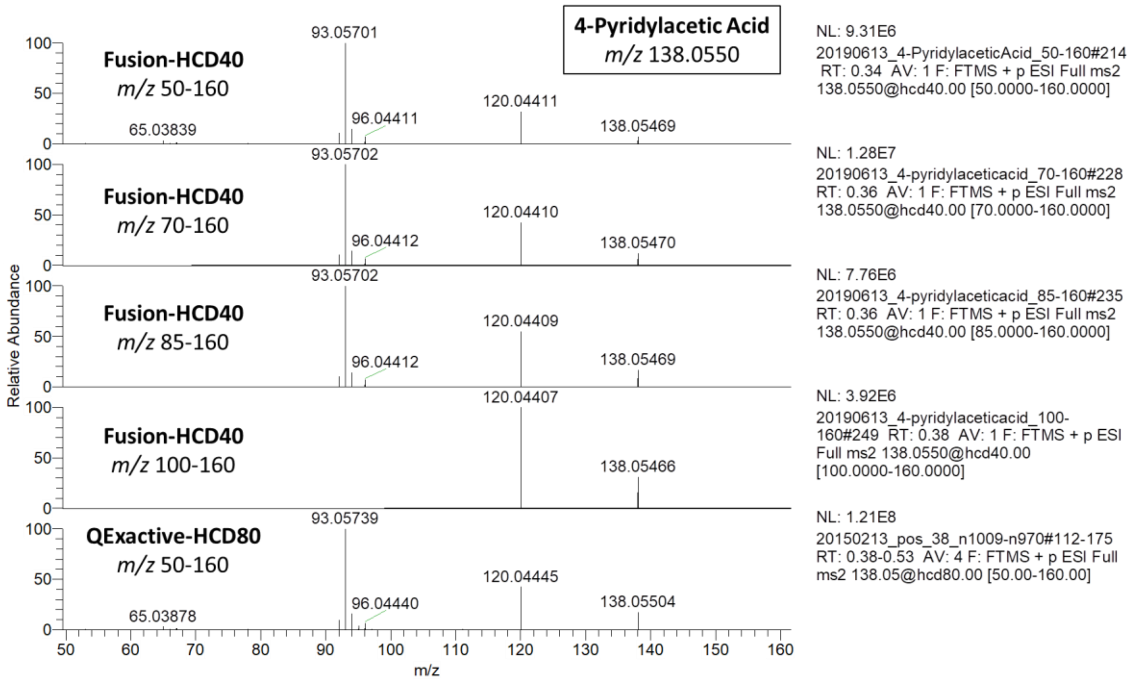

Figure S1. Comparison of HCD spectra acquired for 4-pyridylacetic acid on the Orbitrap Fusion or on a Q-Exactive using variable *m/z* windows for fragment ions.

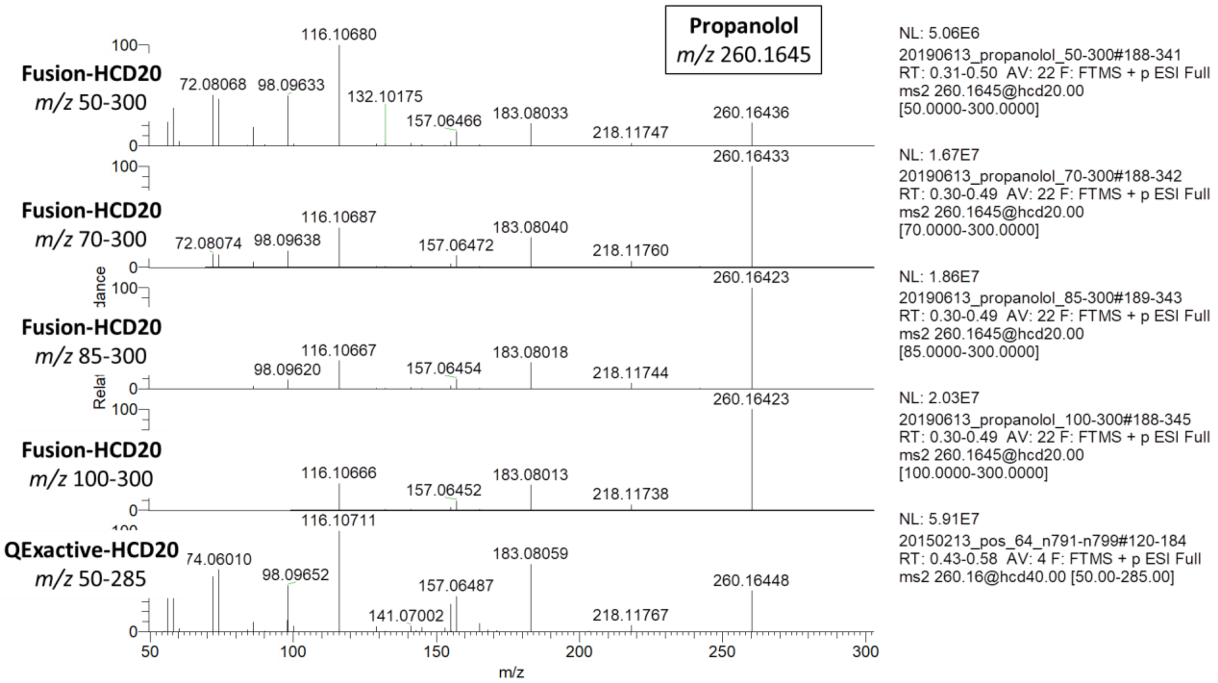

Figure S2. Comparison of HCD spectra acquired for propanolol on the Orbitrap Fusion or on a Q-Exactive using variable *m/z* windows for fragment ions.

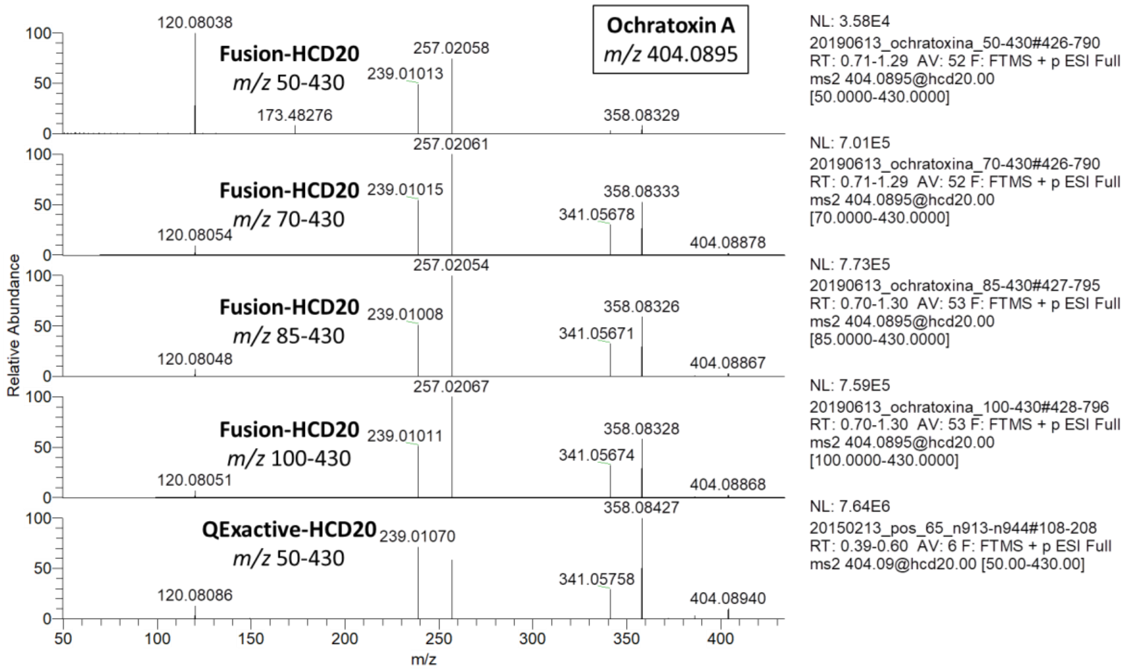

Figure S3. Comparison of HCD spectra acquired for ochratoxin A on the Orbitrap Fusion or on a Q-Exactive using variable *m/z* windows for fragment ions.

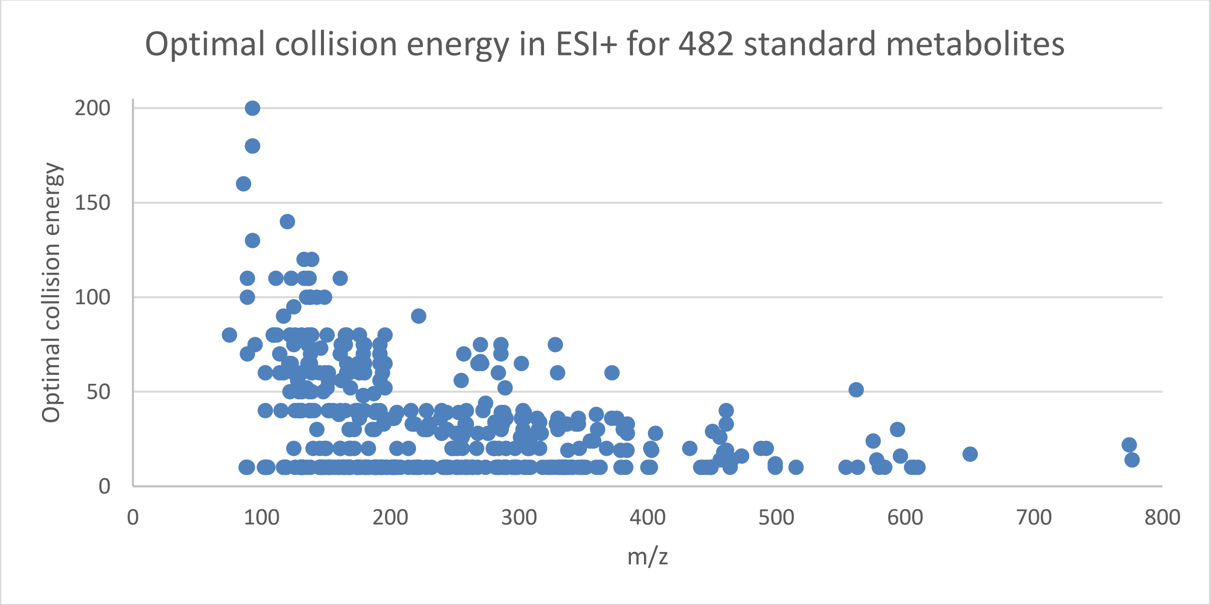

Figure S4. Distribution of optimal collision energy for 482 standard metabolites.
These energies were determined in ESI+ on a Q-Exactive instrument. Collision energy was considered as optimal if the parent ion presents a relative abundance between 15 and 45%.

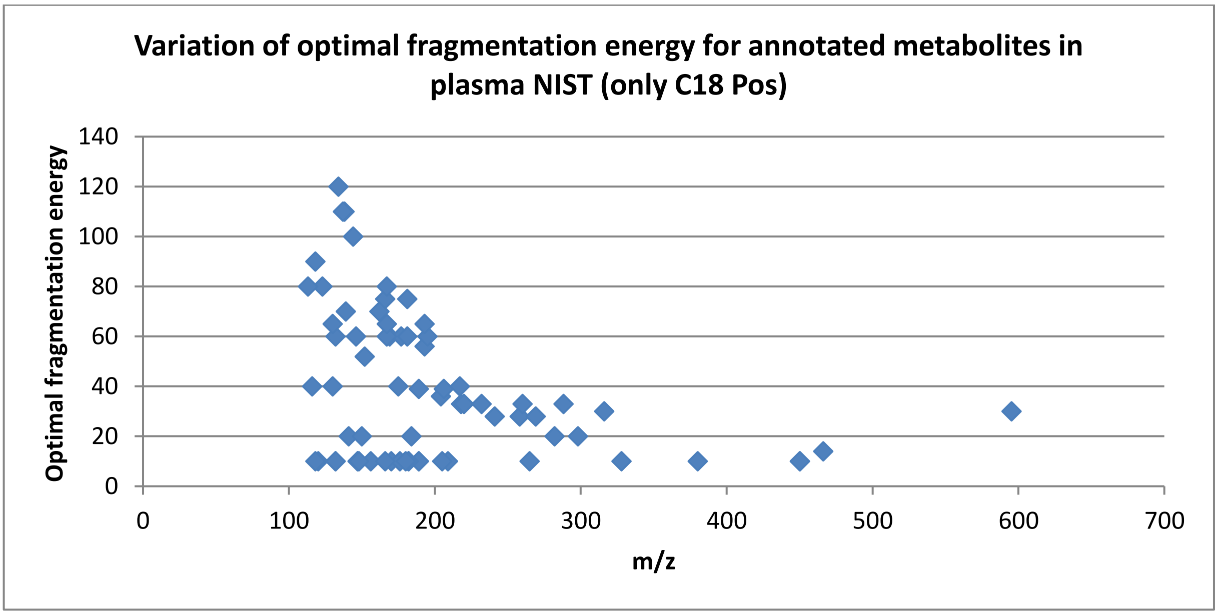

Figure S5. Distribution of optimal collision energy for 72 standard metabolites identified in NIST plasma using C_{18} UHPLC system coupled to (ESI+)-MS/MS.
These energies were determined in ESI+ on a Q-Exactive instrument. Collision energy was considered as optimal if the parent ion presents a relative abundance between 15 and 45%.

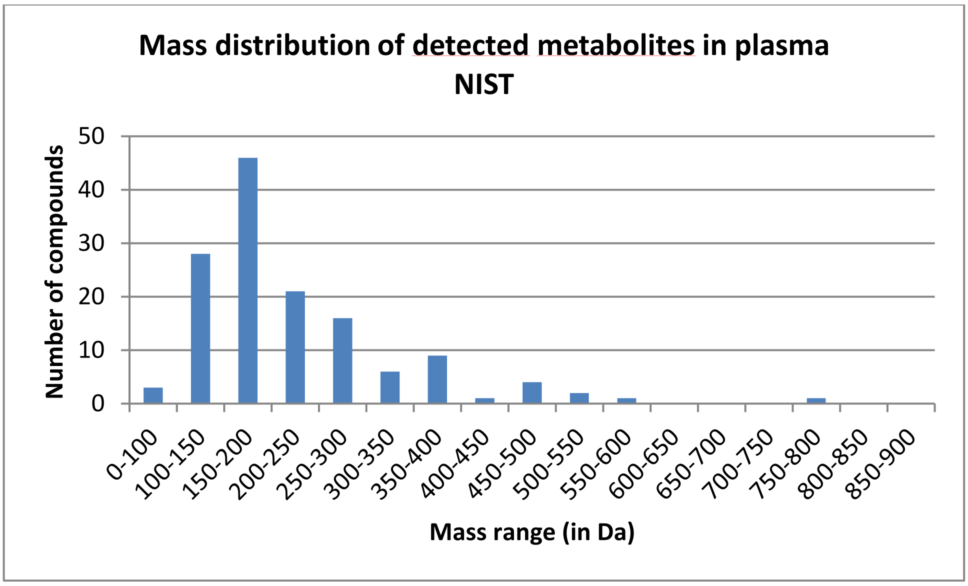

Figure S6. Mass distribution of metabolites identified in plasma.

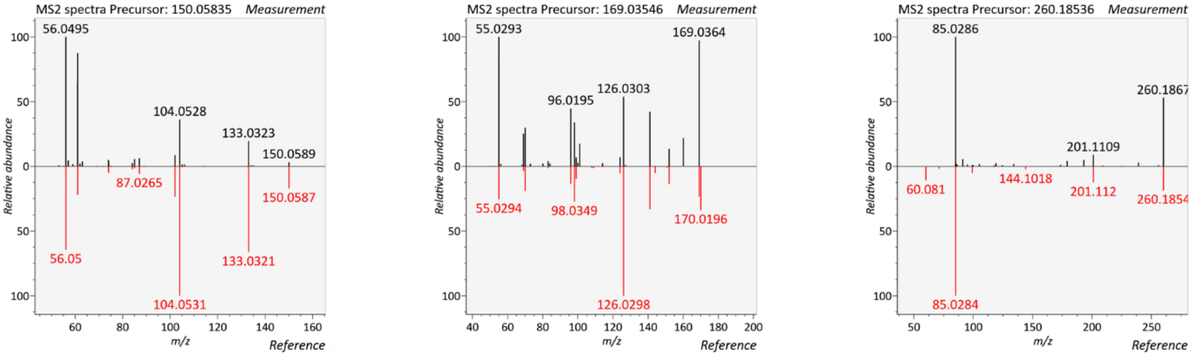

Methionine (DP: 529) **Uric acid** (DP: 690) **Hexanoylcarnitine** (DP: 764)

Figure S7. Head-to-tail comparison of evaluated versus reference MS/MS spectra. Evaluated MS/MS spectra were obtained using the DIA acquisition workflow.

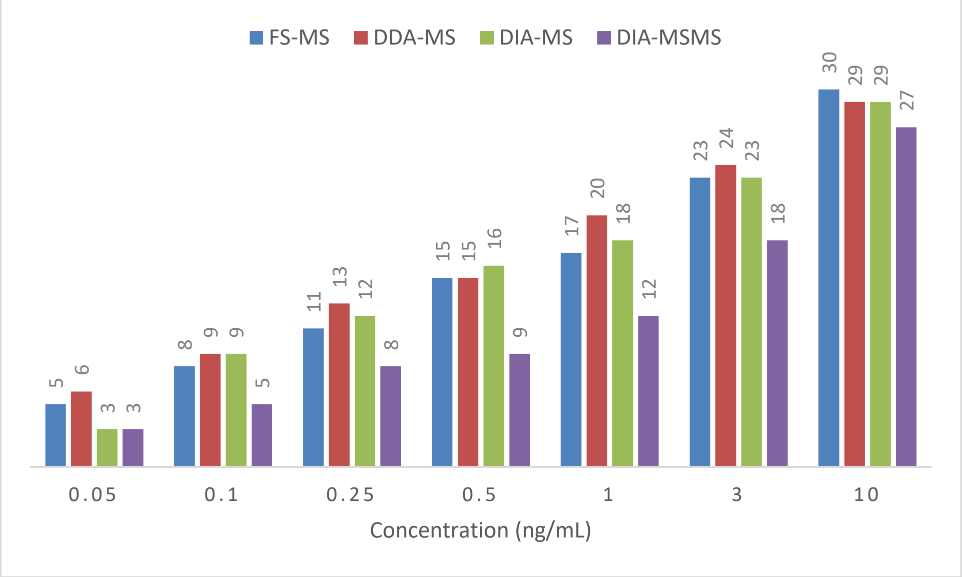

Figure S8. Number of metabolites reproducibly detected (with CV < 30%) using full-scan only (blue), DDA (red), DIA-MS (green) and DIA MS/MS workflows (purple). Each bar is the result of 6 independent measurements. For the molecules from the spiked test mixture also present endogenously in plasma, only signals exceeding more than 5 times the endogenous ones were considered.
